# Broca’s biases in modern aphasiology

**DOI:** 10.3389/fnhum.2026.1783073

**Published:** 2026-04-28

**Authors:** Silvia Martínez-Ferreiro, Monica I. Norvik

**Affiliations:** 1 Gerontology and Geriatrics Research Group, Instituto de Investigación Biomédica de A Coruña (INIBIC), Complexo Hospitalario Universitario de A Coruña (CHUAC), SERGAS, Universidade da Coruña, A Coruña, Spain; 2 Department of Education, UiT—The Arctic University of Norway, Tromsø, Norway; 3 Department of Linguistics and Scandinavian Studies, University of Oslo, Oslo, Norway

**Keywords:** aphasia, assessment, crosslinguistic research, multilingualism, treatment

## Abstract

Broca’s description of aphemia signals the emergence of aphasiology as a discipline. It also marks the beginning of a long tradition of studies of right-handed patients (“Nous parlons avec l’hémisphère gauche”) with lesions in the inferior frontal gyrus (Broca’s area) displaying production deficits (Broca’s aphasia) in their single, mainly Romance or, as years went by, Germanic language. In modern-day aphasiology, language crosses hemispheric boundaries, production is no longer confined to the left inferior frontal gyrus, and lesions in Broca’s area do not necessarily entail Broca’s aphasia, with recognized production deficits associated with other lesion locations and aphasia types. Nonetheless, idealized monolingual speakers are still at the center of most theories and shape the design of clinical materials. In this paper, we address two key questions Broca left unanswered: (1) How well do current linguistic theories accommodate the diversity of typologies in the nearly 7,000 languages of the world? and (2) How adequate are our clinical resources for the assessment and treatment needs of multilingual individuals? After an introduction, we will discuss crosslinguistic contributions to the development of modern aphasiology in section 2. Section 3 will focus on multilingual aphasia studies.

## 1 Introduction

The history of the scientific study of aphasia cannot be told without mentioning the physician, anatomist, anthropologist, and activist Paul Pierre Broca (1824–1880), and the subsequent debates and controversies generated around his figure and his contributions to language localization. Aphasia is an acquired language impairment caused by focal brain damage, leading to difficulties with speaking, understanding, reading, and writing, which severely impact daily life. The use of terms such as Broca’s area and Broca’s aphasia in modern aphasiology stands as a firm testimony of his undeniable historical role. However, exhaustive reviews of early works together with new technological advances in neuroimaging have reopened classical debates and invited scientists to revisit his legacy.

It is generally accepted nowadays that a proper description of the inception of aphasiology as a research field requires recognition of the contributions of a wide variety of personalities throughout history: from the role of Egyptian surgeons through Greco-Roman antiquity to the Renaissance and the observations of the 17th and 18th centuries (for thorough discussion of historical landmarks in the history of aphasia see Code, 2021, 15; LaPointe, 2013; Prins and Bastiaanse, 2006). However, the 19th century played a major role in consolidating the discipline. This was the era of Paul Pierre Broca, but he was far from being alone. (Mostly French) figures such as Aubertin, Bouillaud, Dax, Flourens, and Gratiolet, and the ideas generated in the debates at the *Société d’Anthropologie de Paris* critically advanced our understanding of aphasia through their observations on language localization (LaPointe, 2013). Interestingly, Broca’s (1861, 1865) work was based on two landmark cases, those of M. Leborgne and M. Lelong, which would nowadays be labelled as cases of global aphasia, and whose lesions surpassed the boundaries of Broca’s area (Dronkers et al., 2007; Giménez-Roldán, 2017; Alfredo et al., 2016). The role of Broca’s area and its relationship to Broca’s aphasia has been open to extensive debates in later years. Evidence from patients with Broca’s aphasia without damage to Broca’s area has led to the claim that such lesions would cause only mild or transient language symptoms (Ardila et al., 2016; Fridriksson et al., 2007; Grodzinsky, 2000). We may still speak with the left, “Nous parlons avec l’hémisphère gauche” (Broca, 1865), but bilateral cortical, subcortical, cerebellar areas, neural pathways, and even the cholinergic and the dopaminergic systems have been proven to have an impact on language (Turker et al., 2023), making the outcome of every lesion unique.

Early conceptions with a crucial impact on shaping the field have been gradually abandoned. However, other (subtler) effects derived from early studies have gone largely unnoticed until recent decades and have generated certain biases that are only now being taken into consideration. Broca’s search for language was deeply rooted in the observation of two monolingual French speakers. This would inaugurate a long-lasting tradition of monolingual studies, frameworks, and clinical practices heavily dominated by Western European oral languages, thereby overlooking variation in structures, vocabulary, cultural contexts, and literacy levels (Karanth et al., 1991, 95). It would take almost a quarter of a century after Broca’s foundational work for language structure to be acknowledged (e.g., Baudouin de Courtenay, Ferdinand de Saussure; Fromkin, 2000), and 75 years for the introduction of methodological linguistic tools for data analysis and the development of detailed linguistic profiles of language deficits (Alajouanine et al., 1939; Jakobson, 1941). Shifting the focus to underrepresented and minority languages and multilingual^1^ individuals remains a pressing matter.

The adequacy of current frameworks and the changes that the discipline will necessarily have to undergo to accommodate the world’s linguistic diversity and the crosslinguistic contributions to the development of modern aphasiology are discussed in section 2. Section 3 addresses the adequacy of our clinical resources for the assessment and treatment needs of multilingual individuals.

## 2 Crosslinguistic contributions to the development of modern aphasiology

Research in Romance and Germanic languages has shaped the field of aphasiology from the genesis, representing 89% of aphasia research between 2000 and 2009 (Beveridge and Bak, 2011), with English data being the predominant source of information in the past decades (60.29% papers between 2010 and 2019; Egia-Zabala and Munarriz-Ibarrola, 2024). In a world with more than 7,000 oral and sign languages, organized in roughly 150 families and including language isolates (Eberhard et al., 2025), such a narrow view has hindered our capacity for knowledge building, which crucially depends on crosslinguistic comparability. In a recent study, Tsiwah et al.^2^ found that in the two decades between 2003 and 2023, only 0.7% of the world’s languages were represented in aphasia literature. Moreover, a typological analysis showed that these numbers are not only notoriously insufficient but highly concentrated within a structurally homogeneous region and relatively isolated from broader linguistic diversity. Germanic and Romance languages, the most widely represented branches within the dominant Indo-European family, share a significant amount of linguistic characteristics driven by historical contact. These include, among many others, a predominant use of SVO word order, or V2 (Verb-second) in Germanic languages, reduced complex case systems, or grammatical similarities such as the use of articles (definite/indefinite) to denote noun specificity or a similar structure for compound tenses. Syntactically, more than 40% of the world’s languages are SVO languages (Hammarström, 2016). However, morphologically, agglutinative languages, most notably found in the Uralic, Turkic, Dravidian, Japonic, and Bantu families, are more frequent than the the fusional languages that have shaped many of the theoretical constructs in aphasiology (45% vs. 30% of the world’s languages respectively) (Haspelmath, 2008, 75)^3^. This significantly limits the representativeness of models and theories based on the evidence available to date. Difficulties comparing across typologically different languages undermine our methodological resources to establish control mechanisms, thus restricting the potential for generalizing results across the board (Bates et al., 1991; Ezzedine and Martínez-Ferreiro, 2025, 1; Peña, 2007). Given that the lack of in-depth characterizations of most of the world’s languages has a cascading effect that affects the creation of clinically relevant assessment and intervention tools and the delivery of appropriate treatment (Ali et al., 2021; Badar et al., 2021; Fyndanis et al., 2017; Martinez-Ferreiro et al., 2026), a necessary change of scenario has been emerging in recent years.

### 2.1 Terminology and hypotheses

Inspired by early works such as Menn and Obler’s (1989) *Agrammatic Aphasia: A Cross-Language Narrative Source Book* and Paradis (2001) characterization of 14 new languages, among others, the field has continued to expand its cross-linguistic perspective. Increased data sharing (e.g., AphasiaBank; MacWhinney et al., 2011) and multidisciplinary international collaborations (e.g., The Collaboration of Aphasia Trialists; Arslan and Peñaloza, 2025; Matić et al., 2026), have further accelerated this development. The exhaustive analysis of data from typologically distant languages has led to the updating of terminology, rendering some terms obsolete, and to reconsidering widely accepted theoretical notions (see, e.g., the notion of wordhood itself; Haspelmath, 2011) and hypotheses. Just as aphemia became aphasia (Trousseau, 1864), and Broca’s area became a region or a territory (see, e.g., Grodzinsky and Amunts, 2006), cases such as that of M. Leborgne, the man behind *Tan*, ceased to be described as instances of telegraphic speech, and agrammatism, a term first coined by Kussmaul (1877), is nowadays characterized in terms of reduced grammatical complexity with frequent omissions of free and optional grammatical morphemes. Contrary to earlier statements that focused on the absence of grammatical markers, this redefinition leaves room for languages that express grammatical markers by means of bound elements, which are generally substituted instead of omitted (Grodzinsky, 1990), and languages with simpler grammars, such as Mandarin Chinese, which rely not only on particles but also on word order to express grammatical content. It is thanks to these updates that the traditional agrammatic-paragrammatic distinction, ultimately linked to the grammatical/functional-lexical divide and the classification of non-fluent vs. fluent syndromes, is still in use nowadays despite the progressive abandonment of classic lesion models (Broca vs. Wernicke).

The refinement of terminology is not the only consequence of incorporating new languages and profiles in aphasia studies. The formulation of linguistically elaborated hypotheses has also benefited from the observation of typologically different languages. A prototypical example comes from hypotheses based on movement (e.g., *Trace Deletion Hypothesis*, Grodzinsky, 1986; and *Derived Order Problem Hypothesis*, Bastiaanse and Van Zonneveld, 2006). Whereas the observation of (mostly) Germanic and Romance languages led to the generally accepted notion that constructions such as passives are generally challenging (in non-fluent aphasias) because they entail movement operations that add complexity in comparison to their active counterparts, the study of new datasets has proven that this is not always the case. Jap et al. (2016) provided evidence that in Standard Indonesian, where passives are similarly built through movement operations but are more frequent than in previously studied languages, these tend to be preserved to the same extent as active sentences in people with Broca’s aphasia.

The examples from Mandarin Chinese and Standard Indonesian are just an illustration of the need to explore other typological regions to increase the scope of what has been theorized so far. According to Tsiwah et al. (see text footnote 2), understudied languages in aphasia are embedded within regions of higher syntactic density than the most studied languages. Ignoring these languages jeopardizes our ability to uncover the patterns underlying language breakdown and recovery, thereby compromising the discipline’s ability to respond to or even formulate fundamental questions. Results such as those presented in the preceding paragraphs and similar ones contribute to the perpetual process of contrasting and refining operational hypotheses, promoting the development of the discipline, an effect that extends beyond linguistics to psycholinguistic frameworks (Sung et al., 2024) (see text footnote 2).

### 2.2 Underrepresented and minority languages

Inclusivity has provided additional benefits. The study of signed (vs. oral) languages has allowed us to deepen our understanding of aphasia and its relationship with both language and its neural bases. Results from different sign languages have confirmed a shared neural network and close similarity in language deficits relative to oral languages, with the particularity of modality-specific communication deficits that may emerge from damage to the right hemisphere (Goldberg and Hillis, 2022).

The evolving, more integrative scenario for aphasia research, with a growing number of studies of typologically different languages, has led to the questioning of widely accepted concepts, more caution with generalizations, and increased awareness of the individual profiles and needs of all individuals with aphasia. However, shifting the focus from WEIRD languages (Western, Educated, Industrialised, Rich, and Democratic; Henrich et al., 2010) to include linguistic and cultural diversity in aphasia research and clinical practice has revealed new challenges that need to be urgently addressed. Matić et al. (2026) and Munarriz-Ibarrola et al. (2025) identify key issues that cannot be left outside the research agenda for the aphasiology of the 21st century. Two main points include investigating how aphasia symptoms manifest across linguistic levels in all languages, independent of the number of speakers or modality, and the urgent need to develop culturally sensitive and linguistically relevant tools for minority languages, as well as for majority languages traditionally neglected in aphasia studies. These aims, further developed in the next section, reveal supplemental prerequisites to be met, such as the need to compensate for the existence of limited normative data in minority languages and the need to provide training for Speech and Language Pathologists (SLPs) working in minority-language contexts.

In a recent typological analysis, Tsiwah et al. (see text footnote 2) show that the studied languages remain highly concentrated within a structurally homogeneous region. Multidisciplinarity and international data sharing become crucial at the face of the ambitious enterprise of providing accurate language characterizations, assessment, and treatment for all people with aphasia (PWA), a fundamental right recognized in international agreements like the Convention on the Rights of Persons with Disabilities (CRPD), which supports the right to receive care in one’s primary language (Beveridge and Bak, 2011). Only joint efforts will enable us to strengthen our work with and for them, improve our theories, and make aphasiology a truly global, inclusive discipline (Ezzedine and Martínez-Ferreiro, 2025, 1).

However, accommodating speakers of more than 7,000 languages is not the only challenge aphasiology faces in the 21st century. Although most speakers use multiple languages in their daily lives, to this day, the perspective is still widely monolingual (Egia-Zabala and Munarriz-Ibarrola, 2024). Ignoring the linguistic background of patients or treating them as serial monolinguals, neglects the dynamicity of multilingualism, the role of bidirectional crosslinguistic influences, and many other related factors (e.g., Köpke et al., 2023; Sung et al., 2024), and make us question the adequacy of our clinical resources for the assessment and treatment needs of multilingual individuals, the matter of discussion in the next section.

## 3 Multilingual aphasia studies

Life expectancy and mobility trends across a multilingual world suggest an increase in the number of multilingual people with aphasia (MPWA) for the incoming years (Norvik et al., 2022). Multilingual individuals are not two monolingual individuals in one, with two separate linguistic systems (Grosjean, 1989). Instead, multilingualism is a dynamic phenomenon that evolves and changes throughout the lifespan. The languages of multilingual individuals interact and are shaped by factors such as age of acquisition (AoA), usage patterns, and proficiency in the different languages, and also sociocultural contexts, thus leading to a heterogeneity of profiles across individuals. Variability is one of the most notable characteristics of aphasia, and even more striking in multilingual individuals. Experience variables, linguistic factors such as typological distance between the languages spoken by a given individual, and clinical factors such as lesion location and size, and access to culturally relevant SLP resources, directly affect the impairment and recovery patterns of MPWA (Kuzmina et al., 2019; Russell-Meill et al., 2025).

The shift from a monolingual to a multilingual perspective in aphasiology is motivated by a theoretical and a clinical necessity, as aphasia affects language more than other cognitive domains (Fedorenko and Varley, 2016) and the consequences of a single lesion in the brain may provide a window into multilingual language processing and help refine much needed assessment and treatment protocols.

In brief, current resources for multilingual aphasia cases have improved but remain unevenly adequate, with gaps in tools and treatment methods developed for languages other than English, a lack of cross-linguistic norms, and equitable access. Sections 3.1–3.3 develop this across recovery patterns, assessment, and therapy for multilingual individuals with aphasia.

### 3.1 Recovery patterns in multilingual individuals with aphasia

When a multilingual individual suffers a stroke and acquires aphasia, the different languages may be affected to varying degrees and in different ways. Thus, the aphasia symptoms can vary across languages (Knoph, 2011; Menn et al., 1995). Some decades after Broca’s seminal work, the first descriptions of bilingual aphasia appeared (Pitres, 1983, 26; Ribot, 1887). Ribot and Pitres were the first to suggest that the different languages of MPWA may not recover in the same way. Ribot proposed that the most recent memories are less consolidated and therefore have a higher risk of impairment following a cerebral injury like a stroke. This has been taken to suggest that the first-acquired language (L1) tends to be the most resilient and usually the best preserved following a stroke. This has later been referred to as “Ribot’s law.” Contrasting this, Pitres (1983) suggested that multilingual individuals tended to recover the language that was most familiar to them prior to the injury – that is, the dominant language at the time of the injury (“Pitres rule”). Since then, Paradis has described six different recovery patterns that multilingual individuals may experience following a stroke (Paradis, 1977, 65; Paradis and Libben, 1987). In several studies, he reported that the most frequent of these patterns is *parallel recovery*, that is, when two or more languages simultaneously recover to the same degree, relative to their *premorbid* language status. Other frequent non-parallel patterns are reported to be *selective recovery* – when only one of the languages is recovered – and *successive recovery* – when one language recovers before the other(s). The fact that MPWA may experience selective impairment of their languages even led to the assumption that the different languages were represented in separate brain structures – or even in different hemispheres (Albert and Obler, 1978). This has later been contradicted in several neuroimaging studies (Del Maschio and Abutalebi, 2019, 197; Nadeau, 2019).

Since the early contributions of Ribot and Pitres, a substantial body of research has tried to establish which language(s) of MPWA will be spared following a stroke, and to identify the factors that account for the variability in recovery patterns. Studies reporting parallel impairments have been the most common (e.g., Fabbro, 2001; Paradis, 2001). In a more recent meta-analysis, Kuzmina et al. (2019) examined which language was better preserved in multilingual individuals with aphasia due to stroke (comparing only L1 and L2 for simplicity). Overall, their results corroborated the earlier findings in that L1 was significantly better preserved than L2, indicating differential impairment and providing support to Ribot’s law. They further examined whether variables known to affect language processing, like AoA, language proficiency, language use, and linguistic similarity, may have contributed to this finding. The review showed that AoA played a role. The multilingual individuals who acquired both languages early (before age 7), showed comparable performance in both languages, that is, parallel impairment. Furthermore, when exploring premorbid language proficiency, the results indicated that the individuals in the sample who reported higher premorbid L2 than L1 proficiency (only 4) performed better in that language. The others who had either higher L1 proficiency or equal proficiency in the two languages showed the pattern observed for the sample as a whole: better performance in their L1. Language use and exposure are also known to affect language processing. Therefore, Kuzmina et al. (2019) also examined whether language use influences which language may be better preserved after a stroke. Also this variable modified the overall result; the individuals who had used their L2 more had comparable impairment in their two languages, thus partially supporting Pitres’ rule. Finally, previous reviews have reported that linguistic similarity does not affect the impairment patterns of multilingual individuals with aphasia (Ansaldo and Saidi, 2014), and this was also confirmed by Kuzmina et al. (2019).

The findings from this and earlier studies indicate that several underlying factors account for differential recovery in MPWA, underscoring the importance of considering the language history and linguistic profiles of all languages of multilingual individuals and raising the question of how comprehensive the background questionnaires should be in order to cover all relevant information.

### 3.2 Assessment in multilingual aphasia

#### 3.2.1 Premorbid language history

Given the underlying factors that shape language impairment and recovery in MPWA, it is crucial to establish each individual’s premorbid language history. Because objective records usually do not exist, the most commonly used approach to obtaining this information is via self-ratings to capture the AoA, proficiency, exposure, and use. Detailed questionnaires have been developed to gather information from multilingual individuals with aphasia (e.g., Paradis and Libben, 1987) and for multilingual individuals and without neurological impairments (Kastenbaum et al., 2018; Marian et al., 2007). Although validity and reliability concerns have been reported for self-ratings in healthy populations (Gollan et al., 2012; Tomoschuk et al., 2019), self-rated pre-stroke language skills may predict post-stroke language abilities (e.g., Gray and Kiran, 2013). Nevertheless, many instruments retain a strong monolingual bias, as they assume sequential acquisition of different languages and do not consider the possibility of having more than one L1, and overlook sociolinguistic realities characteristic of multilingual societies (e.g., contexts where the spoken L1 is different from the written L1) (Matić et al., 2026; Multilingual Aphasia Project group of the CATs). Time constraints in clinical settings pose an additional challenge for implementation.

#### 3.2.2 Aphasia assessment tools

Because language acquisition, use, and proficiency differ across languages, and aphasia may affect them differently, it is crucial to assess all the languages of multilingual individuals with aphasia. This assessment should follow a thorough description of the language background. It is also essential to use measures that are both comparable across languages and sensitive to linguistic and cultural differences. To enable valid cross-language comparisons, assessment tools must be equivalent both at the subtest and item levels.

Achieving this is not necessarily straightforward, because aphasiology remains English- and Western-biased. Influential and widely cited diagnostic instruments developed in English are translated or adapted to other languages, often with minimal modifications (e.g., Bates and Wulfeck, 1989; Ivanova and Hallowell, 2013), despite longlasting cautions against the use of translated tests, as they risk overlooking essential linguistic information (Bates and Wulfeck, 1989; Paradis and Libben, 1987). Stimuli may be inappropriate, and linguistic constructions may have different levels of difficulty – or may even be non-existent – in the translated version (Fyndanis et al., 2017; Martinez-Ferreiro et al., 2026). Moreover, most translated tests provide norms in only one language and lack psychometric validation (e.g., validity, reliability; Ivanova and Hallowell, 2013) and thus may be inappropriate for multilingual individuals with diverse language combinations. Nevertheless, English-based tools are the most widely used, and for many languages, existing aphasia tests are not tailored to the language-specific structures of the target language. Using translated non-standardized tests compromises professional standards and may even constitute an ethics violation (Bortz, 2024, p. 6). Clinicians should therefore choose tests that are designed – or at least adequately adapted to—the language in question.

In this context, the 2019 ROMA consensus statement proposed a core outcome set for aphasia treatment studies (Wallace et al., 2019). The *Western Aphasia Battery Revised* (WAB-R; Kertesz, 2006), the *General Health Questionnaire* (GHQ-12; Goldberg and Williams, 1988), and the *Stroke and Aphasia Quality of Life Scale* (SAQOL-39; Hilari et al., 2009) were recommended for assessing language, emotional wellbeing, and quality of life, respectively. Availability in different languages or ease of translation/adaptation was the first feasibility criterion. However, even these widely disseminated tools are yet to be adapted to most languages outside the Indo-European sphere (see Goral and Galletta, 2024, 307 and Ivanova and Hallowell, 2013 for an overview of tests in different languages).

Even for thoroughly controlled, normed, and validated adaptations such as those of the *Comprehensive Aphasia Test* (CAT; Swinburn et al., 2004; Fyndanis et al., 2017; Martinez-Ferreiro et al., 2026), there is a need for comparison studies between the different language versions to ensure equivalence between them. The comparison between the Croatian and Norwegian versions of the CAT (Matić Škorić et al., 2026) showed that the reliability of the two language versions was high and comparable. The study showed that the control groups nearly reached ceiling scores; however, some differences emerged in several of the subtests, with the Norwegian controls and PWA achieving higher scores. The items in the Norwegian version of the CAT thus appear to exhibit lower overall item difficulty, and the authors conclude that the differences between groups can be attributed to variations in language structure, which are also reflected in item difficulty (Matić Škorić et al., 2026).

While none of the aforementioned tests were originally designed to assess language abilities in various languages of multilingual individuals in a comparable manner, other tests, such as the *Bilingual Aphasia Test* (BAT; Paradis and Libben, 1987), were designed to evaluate language abilities across multiple languages. The BAT is available in more than 70 language versions and is normed on neurologically healthy individuals in each language. However, these norms are not published for most languages, dialect variation is unaccounted for (e.g., American Spanish), and few studies have compared the different language versions. Where such comparisons exist, several include small sample sizes. They show that neurologically healthy individuals do not necessarily perform at ceiling, and their scores are modulated by age, education, and language dominance (Guilhem et al., 2013; Juncos-Rabadán, 1994; Manuel-Dupont et al., 1992; Muñoz and Marquardt, 2008). For instance, French-German and French-other-language bilingual individuals showed averages of 95% on the short version in French and German, and a ceiling effect on only a few subtests (Guilhem et al., 2013; Köpke et al., 2015). Older Spanish-Galician individuals performed worse than younger adults (Juncos-Rabadán, 1994), and item analyses of the Spanish-English short version of the BAT found that more than half of the items had a correct response rate below 70%, often due to unfamiliar lexical items (Muñoz and Marquardt, 2008).

Summing up, researchers and clinicians must recognize the potential biases inherent in standardized tests, which are typically based on English and have monolingual norms. The dynamicity of multilingualism and the heterogeneity of multilingual individuals make it difficult to design structured tests appropriate for all MPWA. Combining ecologically valid tasks, such as elicited connected language production using culturally appropriate pictures and narrative production, may help bridge the gap between formal standard assessment and the particularities of the target population, thus leading to more appropriate treatment plans (Norvik et al., forthcoming).

### 3.3 Therapy in multilingual aphasia

Overall, aphasia rehabilitation often focuses on restoring language and communication abilities. Aphasia therapy may be provided with the aim of improving the retrieval of specific words, with an additional goal of improving unassisted word retrieval after the therapy period ends (Howard and Hatfield, 1987). For multilingual individuals with aphasia, the main goal of treatment is to facilitate communication abilities in all the languages needed for participation in meaningful life activities. An overarching goal of aphasia treatment is, obviously, the direct effect of the treatment, as well as its generalization to untreated conditions, tasks, or stimuli. For multilingual individuals, an additional aim is the generalization of treatment gains to untreated languages, also known as cross-linguistic transfer (Goral et al., 2023).

Aphasia treatment must address specific features of the language spoken by the MPWA. As in the case of assessment tools, direct translations of therapy plans across typologically divergent languages might not be appropriate, as they might lead to the design of inadequate treatment strategies based on models tailored for languages different from those spoken by the people we intend to help (Beveridge and Bak, 2011; Quique et al., 2026).

Since the early 2000s, there has been an increase in studies on cross-linguistic transfer in multilingual aphasia (see reviews by Ansaldo and Saidi, 2014; Faroqi-Shah et al., 2010; Kohnert, 2009). The results of the reviews were mixed. Some studies reported cross-linguistic transfer, either from the treated L1 to an untreated L2 or from a treated (weaker) L2 to an untreated L1. Yet other studies failed to achieve cross-linguistic transfer in some participants. Given the equivocal results, an updated meta-analysis was recently published (Goral et al., 2023). The meta-analysis examined whether language therapy in MPWA is beneficial, and which factors influence therapy outcomes, focusing on aphasia-related, treatment-related, and multilingualism-related variables. Overall, the results for improvement within the treated language showed that language interventions for MPWA are beneficial. Post-treatment scores were significantly higher than pre-treatment scores, with larger improvement for trained items than for untrained items, indicating that therapy produced item-specific improvements. This is an important finding, given that most therapy approaches have been developed for monolingual individuals and most of them in English and Western European Languages (Beveridge and Bak, 2011). Regarding multilingualism-related factors, treatment outcomes did not differ when therapy was provided in a first language (L1) versus a non-L1. However, treatment was found to be more effective when provided in an early-acquired language. Neither language proficiency nor aphasia severity influenced within-language treatment effects. For cross-linguistic transfer, however, the effects were weaker. Generalization was observed primarily for translation equivalents of trained words. Among the multilingualism-related variables, AoA emerged as a significant predictor, indicating that cross-linguistic transfer was more likely when treatment was provided in an earlier-acquired language. No effects were found for treatment in L1 versus non-L1, language proficiency, language distance, or aphasia severity.

Overall, language use and exposure stand out as important variables, highlighting the need to review existing policies regarding the treatment of MPWA in favor of personalized, culturally relevant models, advocating for therapy in the patient’s preferred language(s), allowing code-switching, assessing all languages, and using tailored materials to improve outcomes (Imaezue et al., 2025).

## 4 Discussion

Paul Broca was a key pioneer in neuroscience, anthropology, and surgery whose work set the foundations for the study of aphasia. As with any promoter of new ideas, the best course of action to honor his legacy is to ensure that the field he contributed to creating continues to evolve over time. The world of the 21st century is a highly connected world where multiple languages coexist in diverse (and sometimes unequal) ways. In this changing, more integrative scenario for aphasia studies, challenging monolingual views stands as a priority.

With the increase in multidisciplinary international collaborations and the support of technological developments, crosslinguistic data are now more than ever positioned as the key element for identifying core language-general deficits in aphasia and distinguishing them from language-specific patterns while accounting for specific features that may interplay in aphasia (Sung et al., 2024). The immediate consequence is the refinement and improvement of our theoretical resources (e.g., neural bases of linguistic processing, linguistic theory, typological studies), which are critical for ensuring the development of theoretically anchored assessment and treatment protocols.

These protocols should be conceived as tools to meet the needs of all speakers and signers regardless of the language(s) they speak. To do so, factors such as language experience (AoA, pre-stroke proficiency, frequency and context of use), the linguistic characteristics of the spoken languages (typological similarities, code-switching patterns), and clinical factors (lesion site and size, resource availability) must be factored into the designs (Kuzmina et al., 2019).

This new era in aphasia studies, which aligns with general trends toward equitable care and personalized medicine, requires an exercise in self-assessment (*Where are we?*) as well as reflection on the realities surrounding us (*What do we need?*) (Arslan et al., 2026). Broca taught us that we speak with the left, and today we can add that we do so in different languages and, in most cases, in multiple languages. Therefore, to accommodate the current needs of people with aphasia, we must go back to the drawing board, expand our knowledge base by exploring languages that have so far gone unnoticed in international forums, use that fundamental knowledge to create culturally and linguistically appropriate robust tools that facilitate diagnosis and intervention and improve training. At the same time, it involves new players such as interpreters, as well as tools such as AI that enable fluid communication with the real protagonists of aphasiology, each and every person with aphasia.

## Footnotes

1 In this paper, the terms bilingual and multilingual will be used interchangeably and refer to the use of two or more languages (or dialects) in everyday life (Grosjean and Li, 2013).

2 Tsiwah, Lucquiault, V., and Martínez-Ferreiro, S. (Submited). "Canary in the Coal Mine: Mapping the Scientific Costs of Lack of Linguistic Diversity in Aphasia Research.

3 We would like to thank one of the reviewers for the insightful comments on this issue.
